# Birth Weight, Breast Cancer and the Potential Mediating Hormonal Environment

**DOI:** 10.1371/journal.pone.0040199

**Published:** 2012-07-17

**Authors:** Radek Bukowski, Rowan T. Chlebowski, Inger Thune, Anne-Sofie Furberg, Gary D. V. Hankins, Fergal D. Malone, Mary E. D’Alton

**Affiliations:** 1 Department of Obstetrics and Gynecology, University of Texas Medical Branch, Galveston, Texas, United States of America; 2 Los Angeles Biomedical Research Institute at Harbor-UCLA Medical Center, Torrance, California, United States of America; 3 Department of Oncology, Oslo University Hospital, Oslo, Norway; 4 Faculty of Health Sciences, Department of Community Medicine, University of Tromsø, Tromsø, Norway; 5 Department of Obstetrics and Gynecology, Royal College of Surgeons in Ireland, Dublin, Ireland; 6 Department of Obstetrics and Gynecology, Columbia University, New York, New York, United States of America; Sookmyung Women’s University, Republic of Korea

## Abstract

**Background:**

Previous studies have shown that woman’s risk of breast cancer in later life is associated with her infants birth weights. The objective of this study was to determine if this association is independent of breast cancer risk factors, mother’s own birth weight and to evaluate association between infants birth weight and hormonal environment during pregnancy. Independent association would have implications for understanding the mechanism, but also for prediction and prevention of breast cancer.

**Methods and Findings:**

Risk of breast cancer in relation to a first infant’s birth weight, mother’s own birth weight and breast cancer risk factors were evaluated in a prospective cohort of 410 women in the Framingham Study. Serum concentrations of estriol (E3), anti-estrogen alpha-fetoprotein (AFP), and pregnancy-associated plasma protein-A (PAPP-A) were measured in 23,824 pregnant women from a separate prospective cohort, the FASTER trial. During follow-up (median, 14 years) 31 women (7.6 %) were diagnosed with breast cancer. Women with large birth weight infants (in the top quintile) had a higher breast cancer risk compared to other women (hazard ratio (HR), 2.5; 95% confidence interval (CI), 1.2–5.2; P = 0.012). The finding was not affected by adjustment for birth weight of the mother and traditional breast cancer risk factors (adjusted HR, 2.5; 95% CI, 1.2–5.6; P = 0.021). An infant’s birth weight had a strong positive relationship with the mother’s serum E3/AFP ratio and PAPP-A concentration during pregnancy. Adjustment for breast cancer risk factors did not have a material effect on these relationships.

**Conclusions:**

Giving birth to an infant with high birth weight was associated with increased breast cancer risk in later life, independently of mother’s own birth weight and breast cancer risk factors and was also associated with a hormonal environment during pregnancy favoring future breast cancer development and progression.

## Introduction

Prediction of breast cancer plays a pivotal role in its prevention and screening [1]. However, accuracy of traditional methods of breast cancer prediction is limited, principally because not all important risk factors have been identified [2], [3]. Approximately half of the breast cancers can be explained by known risk factors [4]. Recently accumulating evidence suggests that women who had higher birth weight themselves [5] as well as those who had infants with higher birth weight [6], [7] are at higher risk of breast cancer than women who weighted less at birth or who had infants with lower birth weight. However, as there is correlation between a mother’s birth weight and her infant’s birth weights [8], it is unclear whether both are independently associated with the mother’s subsequent breast cancer risk. Although the link to breast cancer for both a women’s own birth weight and the birth weight of her children is biologically plausible, considering great difference in time interval to breast cancer diagnosis, the underlying mechanism is likely different as also would be potential implications for prediction and prevention. Those implications would also be substantial if infant’s birth weight is associated with risk of breast cancer independently of breast cancer risk factors.

During pregnancy a woman is exposed to high concentrations of placenta–derived factors including estriol (E3), anti-estrogen alpha-fetoprotein (AFP) and pregnancy-associated plasma protein-A (PAPP-A)[9]–[11]. The concentrations of E3, AFP and PAPP-A in the maternal circulation during pregnancy are an order of magnitude higher than at any other time during women’s life [9], [12]. While the role of endogenous estrogens in breast cancer development is well established [13], the ratio of the estrogen E3 to the anti-estrogen AFP reflects net estrogenic activity [9], [14]. Moreover, PAPP-A has proteolytic activity targeting insulin-like growth factors binding proteins, which increases concentrations of free insulin-like growth factors [15], [16] and both PAPP-A and insulin-like growth factors are associated with breast cancer development and progression [17], [18]. Infant’s birth weight has been shown to be directly related to maternal concentrations of E3 and PAPP-A and inversely related to those concentrations of AFP [19], [20]. However, those observations could represent a confounding by breast cancer risk factors affecting also both birth weight and hormones concentrations during pregnancy. Breast cancer risk factors weight, height, race and ethnicity, parity and smoking are associated with infant’s birth weight, maternal concentrations of E3, AFP and PAPP-A and are also breast cancer risk factors [21], [22]. Use of fetal growth potential norms to measure infant’s birth weight would allow to account for the potential confounding effect of those variables on the birth weight [23]. Considering these findings, we hypothesized that women’s risk of breast cancer is related to the birth weight of her infant, independently of her own birth weight and breast cancer risk factors and that the association is mediated by an adverse hormonal environment in the maternal circulation reflected by elevated E3/AFP ratio and PAPP-A concentration.

## Materials and Methods

### Study Population

#### Framingham offspring birth history study

Associations between maternal and infant birth weight and subsequent maternal risk of breast cancer were studied in a prospective cohort of women participating in the Framingham Offspring Study of the original participants’ adult children and their spouses. The study design and implementation details were previously described [24]. Briefly, study participants were prospectively followed through 8 examination cycles beginning in 1971. At each visit participants provided a medical history and physical examination and laboratory assessments were obtained. Subjects attending the 5^th^ and 6^th^ examination cycle (1991–1998) and who also participated in the Framingham Offspring Birth History Study were eligible for inclusion in the current analysis. Among 509 women participants, 426 had a live birth with infant birth weight available in 419. Maternal birth weight was also known in 235 of those women. All participants provided written informed consent and the study was approved by the Institutional Review Board.

#### FASTER trial

Associations between infants birth weight and maternal serum concentrations of E3, AFP and PAPP-A were studied in a prospective cohort of women participating in the First and Second Trimester Evaluation of Risk for Aneuploidy (FASTER) trial. Study design and implementation details have been previously described [25]. In brief, the FASTER trial was a prospective observational study performed in 15 U.S. clinical centers between 1999 and 2003. The study participants were women 16 years of age or older with singleton pregnancy recruited in the first trimester of pregnancy. These women had a measurement of PAPP-A in the first trimester and of E3 and AFP in the second trimester using standard assay techniques at a central laboratory (Women’s and Infants Hospital, Rhode Island). Analyses included 23,824 women who gave birth to singleton live infants without chromosomal or structural abnormalities and had complete data on maternal age, height, weight, race/ethnicity, parity, education, marital status, alcohol intake, smoking status and reproductive technologies assisted conception, the factors affecting infants birth weight as well as risk of breast cancer. Institutional Review Board approval was obtained from all sites and all participants gave informed consent.

### Birth Weight

Participants in the Framingham Offspring Birth History Study provided information on their own birth weight and the birth weight of their children, along with other factors related to those births. For this analysis we evaluated the birth weight of the first live born infant. Collected was also information on breast cancer risk factors including age, age at menarche, age at first live birth, age at menopause, race/ethnicity, parity, body mass index, diabetes and use of hormone replacement therapy. Maternal history of breast cancer was determined among women whose mothers participated in the Framingham cohort.

In the FASTER trial infants birth weight was recorded at birth and study analyses were conducted using birth weight itself and using growth potential norms. Growth potential norms account for physiologic and pathologic factors affecting fetal growth including gestational age, maternal weight, height, race/ethnicity, parity, education, marital status, smoking status and reproductive technologies assisted conception, characteristics associated with both birth weight and risk of breast cancer [23]. The growth potential norms were not adjusted for maternal concentrations of studied hormones.

### Outcomes

In the Framingham Offspring Birth History Study breast cancer definition included invasive and non-invasive breast cancer. At each study visits, information on clinical outcomes including cancer was collected. All cancer reports were verified by central medical record review and review of submitted pathology specimens. All cancer cases had microscopic confirmation.

In the FASTER trial analyses serum concentrations of E3, AFP and PAPP-A were measured using standardized immunoassays (Diagnostic Systems Laboratories Inc, Webster, TX and Diagnostic Products Corporation, Los Angeles, CA). Results were converted to multiples of the gestational age medians (MOM) and corrected for maternal weight.

### Statistical Analysis

The associations between maternal and infant birth weights and maternal risk of breast cancer in the Framingham Offspring Birth History Study cohort were analyzed using multivariable proportional-hazards models. Age was used as the time scale, age at examination as time of study entry and breast cancer diagnosis was taken as the event. The loss to follow-up or death unrelated to breast cancer were censored. Cumulative probability of breast cancer curves were constructed with the use of the Kaplan–Meier method for subjects with infants birth weights in top and in lower quintiles.

Hazard ratios for breast cancer were calculated for the infant birth weight in the top quintile compared to the lower quintiles using proportional hazard regression. The hazard ratios were adjusted for the mothers own birth weight and propensity score. The propensity score was calculated using logistic regression to account for the influence of breast cancer risk factors which could also be associated with infant’s birth weight and potentially confound the association. The factors included in the propensity score were age at entry into the study, age at menarche, age at first live birth, age at menopause, race/ethnicity, parity, body mass index, diabetes at entry into the study, family (participants mothers) history of breast cancer and use of hormone replacement therapy [26]. Non-linearity of the propensity score components was tested for using multivariable fractional polynomial regression [27], [28]. The missing values of covariates were replaced using multiple imputations method recommended for time to event data [29]. Proportional hazard assumption was evaluated using global and specific tests [30].

The associations between infant birth weight and growth potential percentile and maternal serum E3/AFP ratio and concentrations of PAPP-A were evaluated using multivariable fractional polynomial and logistic regressions [27], [28]. The associations were adjusted for gestational age and for maternal age, height, weight, race/ethnicity, parity, education, marital status, alcohol intake, smoking status and reproductive technologies assisted conception, the covariates associated with the birth weight and the risk of breast cancer. Goodness of fit was assessed using test of Hosmer and Lemeshow. All p values were two-sided, and p values less than 0.05 were considered to indicate statistical significance. Statistical analyses were performed using Stata 11 (Stata Corporation, College Station, TX, USA).

## Results

In the Framingham Offspring Birth History Study participants of 419 women who had a live birth, four did not attend subsequent examinations and five had a prior or concomitant breast cancer diagnosis. Characteristics of the remaining 410 women included in study analyses are shown in Table 1.

**Table 1:** Baseline Characteristics of the Framingham Offspring Birth History Study Participants.

Characteristic		
	N (%)	Mean ± SD or n (%)
Age - yr	410 (100)	54 ±9
Weight - kg	407 (99)	70.8 ± 15.1
Height - cm	407 (99)	160 ± 6
Race/ethnicity	347 (85)	
White		308 (89)
Hispanic		39 (11)
Age of menarche - yr	372 (91)	13 ± 2
Age of menopause - yr	378 (92)	45 ± 8
Age of first live birth - yr	408 (99)	24 ± 4
Maternal breast cancer	399 (97)	
Yes		38 (10)
No		361 (90)
HRT	362 (88)	
Yes		37 (10)
No		325 (90)
Birth weight infant - g	410 (100)	3304 ± 569
Birth weight mother - g	232 (57)	3267 ± 642

N (%), is a total number and proportion of non-missing data for each characteristic. All characteristics are described as mean +/- SD or as numbers and proportions of participants within categories of the characteristic, n (%). Age, age at menopause, weight, height, use of hormone replacement therapy (HRT) are those at the entrance into the study.

**Figure 1:**
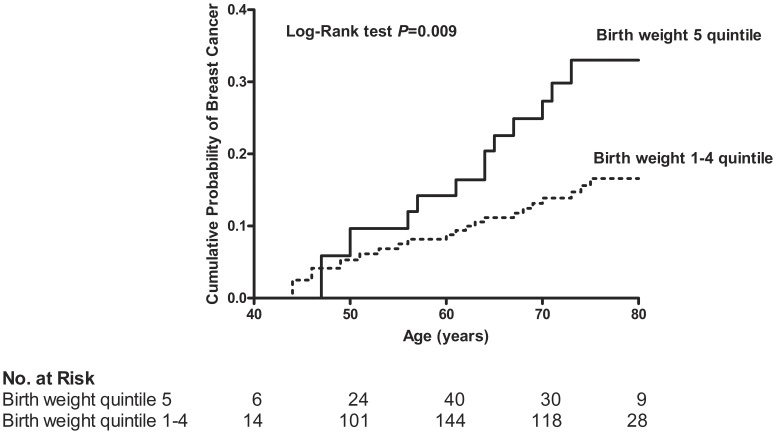
Kaplan–Meier Curves of the Cumulative Probability of Breast Cancer According to Infant Birth Weight Quintile.

During up to 17 years of observation (median 14, interquartile range 13–15 years) 31 of 410 women (7.6 %) were diagnosed with breast cancer. The median time interval from delivery of the first child to diagnosis of breast cancer was 38 years (interquartile range 29–44 years). The median age at the diagnosis was 61 years (interquartile range 55–67 years). Women with large birth weight infants (in the top quintile) had significantly higher risk of breast cancer than women with infants whose birth weights were in lower quintiles (Log-rank test p = 0.009). The cumulative probability of breast cancer for women whose infant’s birth weights were in the top compared to lower quintiles are depicted by the Kaplan-Meier curves (Figure 1).

In the time-to-event analysis the risk of breast cancer was 2.5 fold higher in women whose infant’s birth weight was in the top quintile compared to women with infant birth weight in the lower quintiles (HR 2.5; 95% CI 1.2–5.2, P = 0.012) (Table 2). The risk of breast cancer was not significantly associated with the birth weight of the mother (HR 2.0; 95% CI 0.7–5.8, P = 0.2) and this HR was attenuated when adjusted for the birth weight of the mother’s infant (HR 1.5; 95% CI 0.5–4.5, P = 0.5). The association between giving birth to an infant with large birth weight and a mother's risk of breast cancer was essentially unchanged by adjustment for the mother's own birth weight (HR 2.8; 95% CI 0.98–7.9, P = 0.054) (Table 2). In this subset of women with non-missing mothers’ and infants’ birth weights the association between large mother’s birth weight and her risk of breast cancer was weaker and non-significant (HR 1.5; 95% CI 0.5–4.5, P = 0.5) (Table 2). Analyses with missing mothers' birth weights imputed were essentially unchanged. In those analyses the infant's birth weight was associated with mother's risk of breast cancer (HR 2.5; 95% CI 1.2–5.3, P = 0.011) and remained significant after adjustment for mother’s own birth weight (HR 2.2; 95% CI 1.03–4.8, P = 0.042). In analysis with missing mothers’ birth weights imputed mother’s birth weight was not significantly associated with her risk of breast cancer (HR 2.2; 95% CI 0.9–5.5, P = 0.09) and this association was attenuated by adjustment for infant’s birth weight (HR 1.9; 95% CI 0.7–4.7, P = 0.2). Adjustment for propensity score of breast cancer risk factors also did not have a substantial effect on these relationships (Table 2). The risk of breast cancer in relation to infant’s birth weight was not significantly different among women with large own birth weight (incidence rate ratio 2.2; 95% CI 0.3–26.7) and women with own birth weight that was not large (incidence rate ratio 3.2; 95% CI 0.7–12.6; Mantel-Haenszel test of homogeneity, p = 0.7).

**Table 2:** Risk of Breast Cancer in Relation to Infants’ and Mothers’ Birth Weights.

	Risk of Breast Cancer				
	Unadjusted		Adjusted**		
	Hazard Ratio(95% CI)	P	Hazard Ratio(95% Cl)	P	
**Birth weight** *					
**Infants^1^**	2.5 (1.2–5.2)	0.012	2.9 (1.4–6.1)	0.006	
**Mothers^2^**	2.0 (0.7–5.8)	0.2	2.3 (0.9–6.1)	0.1	
**Infants^3^**	2.8 (0.98–7.9)	0.054	2.5 (1.2–5.6)	0.021	
**Mothers^3^**	1.5 (0.5–4.5)	0.5	2.0 (0.8–5.2)	0.2	

* Presented are three associations between the risk of breast cancer and the birth weights (top quintile vs. lower quintiles) of: the infant^1^, the mother^2^ and both^3^ birth weights.

** Hazard ratios were adjusted for propensity score accounting for: age, age at menarche, age at the birth of the first child, age at menopause, maternal history of breast cancer, parity, body-mass index, race/ethnicity, diabetes and use of hormone replacement therapy. All covariates were measured or reported at the entry into the study. The adjusted analyses were carried out on data with missing covariates values for mother’s own birth weight and propensity score imputed using multiple imputation.

The analyses of proportional hazard assumption were performed for all comparisons presented in subsets of subjects without missing data. All proportional hazard assumption tests showed no evidence of a significant non-proportional hazard (p>0.05, for all).

**Figure 2:**
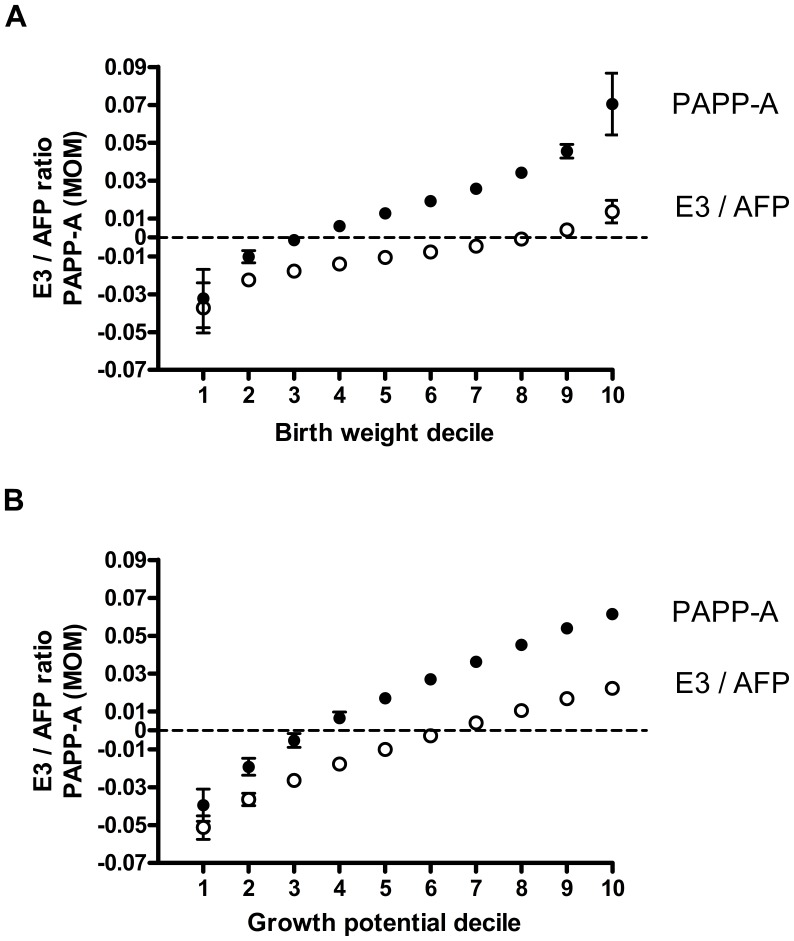
Association between size at birth and maternal serum estriol to alpha-fetoprotein ratio (E3/AFP) and pregnancy-associated plasma protein-A (PAPP-A) concentrations. Maternal serum concentrations of PAPP-A and ratio of E3/AFP (mean +/- SD of log _10_ multiples of the median, MOM or of log _10_ of ratio of MOMs, respectively) plotted against deciles of birth weight (A) or growth potential (B) at birth predicted from fractional polynomial regression analysis. Regression equations: log E3/AFP  =  −.0046098 + .0889807(gp^0^.^5^ −.6792581776); log PAPP-A  =  .0245553 + .1220838(gp^0^.^5^–.6792581776); P<0.0001 for both. (gp  =  growth potential) log E3/AFP  =  −.0090871+ .0000268(bw-3365.677323); log PAPP-A  =  .0160142 + .0084278((bw/1000)^2^-11.32778384); P<0.0001 for both. (bw  =  birth weight) Adjustment for maternal age, height, weight, race/ethnicity, parity, education, marital status, alcohol drinking and smoking status and conception assisted by reproductive technologies did not have a material effect on coefficients for birth weight or growth potential. Dashed line denotes median PAPP-A concentration or E3/AFP ratio of 1.

The characteristics of the study participants from the FASTER trial cohort are presented in Table 3. Among 23,824 pregnancies, a strong positive relationship was observed between the infant birth weight, both unadjusted and percentile of individual growth potential, and the mother’s serum E3/AFP ratio and serum PAPP-A concentration (Figure 2). Adjustment for covariates associated with risk of breast cancer did not have a material effect on those associations (p<0.0001, for all).

**Table 3:** Baseline Characteristics of the FASTER trial Participants.

Characteristic	
	Mean + SD or n (%)
Age - yr	31 ± 6
Weight - kg	67.1 ± 14.3
Height - cm	165 ± 7
Race/ethnicity	
White	16,993 (71)
Hispanic	4,689 (20)
Black	979 (4)
Asians	961 (4)
Other	202 (1)
Parity	
Nulliparous	11,098 (47)
1 or 2	11,030 (46)
3 or 4	1,502 (6)
≥5	194 (1)
Education – yrs.	14 ± 2
Marital status	
Single	3,994 (17)
Married	19,583 (82)
Other	247 (1)
Smoking	
Yes	870 (4)
No	22,954 (96)
Alcohol use	
Yes	510 (2)
No	23,314 (98)
ART	
Yes	1,047 (4)
No	22,777 (96)
Birth weight - g	3,366 ± 534
Gestational age – wks.	39.3 ± 1.9

All characteristics are described as mean +/− SD or as numbers and proportions of participants within categories of the characteristic, n (%). The mother’s age is the age at the time of delivery, and the weight, height, smoking status and alcohol use are those documented at her enrollment into the study in the first trimester of pregnancy.

ART, assisted reproduction technologies is conception assisted with ovulation induction.

Gestational age, is gestational age at delivery in days based on first trimester ultrasound dating.

Women whose infant’s birth weight was in the top quintile had a 25% increased risk of having an E3/AFP ratio in the top quintile (adjusted odds ratio, 1.25; 95%CI, 1.16–1.36; P<0.001) and a 25% increased risk of having a PAPP-A concentration in the top quintile (adjusted odds ratio, 1.25; 95%CI, 1.16–1.35; P<0.001), in comparison to other women. Women with infant growth potential percentile in the top quintile had a 30% increased risk of having an E3/AFP ratio in the top quintile (adjusted odds ratio, 1.30; 95%CI, 1.20–1.41; P<0.001) and a 35% increased risk of having a PAPP-A concentration in the top quintile (adjusted odds ratio, 1.35; 95%CI, 1.25–1.45; P<0.001), in comparison to other women. Goodness of fit test did not show evidence of poor fit (p>0.05 for all).

## Discussion

We investigated the relationship between a woman’s risk of breast cancer and her own birth weight and the birth weight of her first child. In a separate cohort we also evaluated the relationship between an infant’s birth weight and the hormonal environment during pregnancy. We observed that women giving birth to large birth weight infants were at increased risk of subsequent breast cancer. This risk was independent of the mother’s own birth weight and traditional breast cancer risk factors. In a separate analysis, mothers whose infants had large birth weights had during pregnancy, elevated E3/AFP ratios and higher PAPP-A concentrations, hormonal conditions favoring breast cancer development and progression.

A recent re-analysis of individual participant data from 32 studies and meta-analyses found that a woman’s birth size correlated with subsequent breast cancer in adulthood [5], [31], [32]. Other studies reported that women who had infants with higher birth weight were at greater breast cancer risk [6], [7]. However, as a woman’s birth weight and the birth weight of her children are correlated, they could mutually confound their relationships with breast cancer [8]. Findings of this study suggest that women who give birth to high birth weight infant are at increased risk of breast cancer independent of women’s own birth weight. In this analysis only a relatively weak correlation between maternal and infant birth weight was observed (r^2^ = 0.20, P = 0.002), a finding nearly identical to one reported in a large population of over 100,000 families [8]. Limited sample size precluded reliable determination of the association between a woman’s own birth weight and her subsequent breast cancer risk. However, the effect of maternal own birth weight on the risk of breast cancer in this population was smaller than that of the woman’s infant’s birth weight and was not significant either before or after adjustment for breast cancer risk factors. In the model including both maternal and infant birth weights, the hazard ratio for large maternal birth weight decreased after accounting for infant birth weight, while hazard ratio for large infant birth weight remained essentially unchanged by adjustment for maternal birth weight. The analysis of data with missing mothers’ own birth weights imputed also has shown that infant’s birth weight is related to mother’s risk of breast cancer after adjustment for mother’s own birth weight. This finding together with the evidence that women who do and do not recall their birth weight do not differ in their recorded birth weights, suggest that the findings are unlikely the result of confounding by missing mothers’ birth weights [33]. The risk of breast cancer in relation to infant’s birth weight was not significantly different among women with and without large own birth weight. However, the number of breast cancer events in those strata was small and interpretation of this finding has to be cautious. The association between large infant birth weight and breast cancer was also essentially unaffected by adjustments for traditional breast cancer risk factors. The variables included in the propensity score are potential confounders related to both the birth weights and the risk of breast cancer[6], [23], [34]–[36]. Inclusion of hormone replacement therapy is due to predisposition of women with high body mass index to menopausal symptoms and thus use of hormone replacement as well as to giving birth to large birth weight infants [37]. The adjustment included also the BMI and diabetes accounting for the potential confounding effect of those factors [35]. Even without adjustment the effect of those confounders would be expected to be much smaller than observed association.

Since after the first pregnancy the effect of infant birth weight on the maternal risk of breast cancer is likely to be confounded by the subsequent pregnancies, we evaluated the effect of the first infant’s birth weight [38]. Subsequent pregnancies would affect risk of breast cancer by their birth weight related hormonal environment but also through modifying effect of increasing parity. Furthermore, small number of exposures, women with multiple large birth weight infants and even smaller number of breast cancers among those women precludes inferences about the effect of giving birth to multiple large birth weight infants on the risk of breast cancer. Delivery of the first infant preceded diagnosis of breast cancer by median of over 30 years. Thus, giving birth to an infant with a large birth weight is a breast cancer risk factor preceding diagnosis by decades, potentially providing opportunity for early diagnosis and for prevention measures such as breastfeeding, lifestyle modifications or chemoprevention[39]–[43].

In this study we have shown, that women delivering large babies have elevated E3/AFP ratio and PAPP-A concentrations, thus likely high net estrogen activity and free insulin-like growth factors concentrations, both favoring breast cancer development and growth. The association with the percentile of growth potential was stronger than with the infants birth weight, likely due to lesser degree of confounding by maternal characteristics associated both with the birth weight and with the risk of breast cancer.

It is biologically plausible that the relationship between delivery of a large infant and increased risk of breast cancer is mediated by the hormonal environment during pregnancy of high maternal concentrations of E3, low concentrations of AFP and high concentrations of PAPP-A. The circulating concentrations of those hormones during pregnancy are massive in relation to other times in woman’s life [12], [44], [45]. The role of endogenous estrogens in breast cancer development is well established [13] and AFP has anti-estrogenic activity due to binding of estrogen and their receptor [11]. Low AFP concentrations during pregnancy have been associated with increased risk of subsequent breast cancer [14]. PAPP-A is produced by placental trophoblast and has proteolytic activity against insulin-like growth factors binding proteins, increasing concentrations of free insulin-like growth factors [15], [16]. Both, PAPP-A and insulin-like growth factors are potentially involved in breast cancer development [17], [18]. Thus, an elevated ratio of estrogen E3 to anti-estrogen AFP and PAPP-A concentrations in the circulation of women with large infant birth weight would expose those women during pregnancy to a hormonal environment of elevated net estrogenic activity and higher concentrations of free insulin-like growth factors. Recently, preclinical studies have suggested that breast stem cells population may expand or contract, retaining a “memory” of prior hormone exposure [46] providing a potential mechanism for long term effects on breast cancer risk of such exposures during pregnancy. Such exposure could explain the increased risk of breast cancer seen following pregnancy especially in women with large infant birth weight.

A limitation of this study is that the association between large infants birth weight and risk of breast cancer and the association between large infants birth weight and a hormonal environment during pregnancy were tested in different populations. Thus, we were not able to definitely demonstrate that hormonal environment during pregnancy mediates the relationship between large infants birth weight and the risk of breast cancer. However, observation of complementary findings in two different populations would be unlikely if the findings were spurious. The birth weights in the Framingham Birth History Study were self-reported but maternal recall was shown to be highly reliable for first births [47]. Recall of woman’s own birth weight is less accurate, however it was shown to have good validity for high birth weight category [33]. Thus, self report of the birth weight is unlikely to result in a significant misclassification in this study. Almost half of the mothers’ birth weights were not available limiting inference about their effects on their risk of breast cancer. However, this limitation is unlikely to affect the study findings because analysis in a subset of women with non-missing mothers’ and infants’ birth weights showed a strong association between infant’s birth weight and maternal risk of breast cancer and weaker and non-significant association between mother’s birth weight and her risk of breast cancer. Moreover, the results of analyses with and without missing mothers’ birth weights imputed showed very similar findings.

In summary, delivery of a large birth weight infant is a strong and independent risk factor for later breast cancer in women, which precedes the cancer diagnosis by decades. These results suggest that a pregnancy that produces a large birth weight infant is associated with a hormonal environment in the mother favoring breast cancer development and progression and results in increased risk of breast cancer following this pregnancy.
